# Residents' Attitude, Knowledge, and Perceived Preparedness Toward Caring for Patients from Diverse Sociocultural Backgrounds

**DOI:** 10.1089/heq.2016.0010

**Published:** 2017-02-01

**Authors:** Jessie Kimbrough Marshall, Lisa A. Cooper, Alexander R. Green, Amanda Bertram, Letitia Wright, Niki Matusko, Wayne McCullough, Stephen D. Sisson

**Affiliations:** ^1^ Department of Medicine, University of Michigan Health System, Ann Arbor, Michigan.; ^2^ Department of Medicine, Johns Hopkins University School of Medicine, Baltimore, Maryland.; ^3^ Department of Medicine, Massachusetts General Hospital, Boston, Massachusetts.; ^4^ Office of Health Equity and Inclusion, University of Michigan Health System, Ann Arbor, Michigan.; ^5^ Division of Public Health, College of Human Medicine, Michigan State University, East Lansing, Michigan.

**Keywords:** cross-cultural care, graduate medical education, resident training, vulnerable populations

## Abstract

**Purpose:** Training residents to deliver care to increasingly diverse patients in the United States is an important strategy to help alleviate racial and ethnic disparities in health outcomes. Cross-cultural care training of residents continues to present challenges. This study sought to explore the associations among residents' cross-cultural attitudes, preparedness, and knowledge about disparities to better elucidate possible training needs.

**Methods:** This cross-sectional study used web-based questionnaires from 2013 to 2014. Eighty-four internal medicine residency programs with 954 residents across the United States participated. The main outcome was perceived preparedness to care for sociocultural diverse patients.

**Key Results:** Regression analysis showed attitude toward cross-cultural care (beta coefficient [β]=0.57, 95% confidence interval [CI]: 0.49–0.64, *p*<0.001) and report of serving a large number of racial/ethnic minorities (β=0.90, 95% CI: 0.56–1.24, *p*<0.001), and low-socioeconomic status patients (β=0.74, 95% CI: 0.37–1.10, *p*<0.001) were positively associated with preparedness. Knowledge of disparities was poor and did not differ significantly across postgraduate year (PGY)-1, PGY-2, and PGY-3 residents (mean scores: 56%, 58%, and 55%, respectively; *p*=0.08).

**Conclusion:** Residents' knowledge of health and healthcare disparities is poor and does not improve during training. Residents' preparedness to provide cross-cultural care is directly associated with their attitude toward cross-cultural care and their level of exposure to patients from diverse sociocultural backgrounds. Future studies should examine the role of residents' cross-cultural care-related attitudes on their ability to care for diverse patients.

## Introduction

Cross-cultural care training has been identified as an important strategy to help healthcare providers deliver higher quality care to socioculturally diverse patients.^1^ Sociocultural factors include race, ethnicity, primary language, income, education, religion, and other customs and values that characterize a group.^2^ These characteristics can play a role in shaping patient's beliefs, perceptions, and health behaviors.^3^ Health communication and clinical decision-making can be adversely affected, when patient and providers have differences in their sociocultural backgrounds (e.g., differences in English proficiency resulting in language barriers).^4^ Cross-cultural care involves the ability to provide quality healthcare and effectively communicate with diverse patients.^5^ More than a decade ago, the Institute of Medicine released two landmark reports, Crossing the Quality Chasm and Unequal Treatment, which heightened the awareness of the critical role of cross-cultural care in improving quality and eliminating healthcare disparities.^1,6^ Yet, racial and ethnic healthcare disparities persist and will magnify as the diversity of the U.S. population continues to grow. Factors contributing to disparities at the provider level have included limited cross-cultural care skills, bias, and stereotyping.^1^ The changing U.S. demographics drive the need for trained physicians able to deliver high-quality care to socioculturally diverse patients.^1,7–9^

Residency training is an important opportunity to train resident physicians on high-quality cross-cultural care, including educational content on health and healthcare disparities.^10^ Previous studies of residents have shown them to not feel well prepared to provide cross-cultural care with associated factors, including limited training and role modeling.^11,12^ The Accreditation Council for Graduate Medical Education now mandates cross-cultural care training and evaluation for residents.^13^

We hypothesized that residents were poorly trained on contributors to healthcare disparities, and that their self-rated knowledge of healthcare disparities was higher than their measured knowledge. In addition, we posited that a curriculum on the definition of terms used to describe healthcare disparities could improve resident knowledge. We also believed that such a curriculum was only one step in addressing healthcare disparities, and that further studies will be needed to determine its impact on actually improving healthcare disparities. Overarching to our curriculum and this study is the Branch and Fraser conceptual framework model of cultural competency, which postulates that cross-cultural care training can potentially reduce racial and ethnic health disparities at the healthcare provider level.^14^

In this study, we explore the association of resident physicians' self-reported preparedness to care for diverse sociocultural patients and their attitude toward cross-cultural care, in addition to knowledge of racial and ethnic health and healthcare disparities. Other explored variables included resident level of training, patient practice composition, and residency program characteristics.

## Methods

### Study design

We conducted a cross-sectional study of data collected from a cross-cultural care training module administered to internal medicine residents from July 1, 2013, to June 30, 2014. This e-learning module on health and healthcare disparities was written based on established concepts of curriculum development^15^ and disparities in health outcomes.^1,7,10,16^ Multiple choice (pre- and post-test) questions were developed based on the module content. Also, a self-assessment survey was developed using items from validated instruments on attitudes about cross-cultural care, preparedness to care for diverse patient populations, and behaviors in delivering care to culturally diverse patients.^11,17^ The self-assessment survey and pretest questions were used in the study's analysis.

### Study sample

The didactic module was used by residents who subscribed to the Johns Hopkins Internal Medicine Ambulatory Curriculum (www.peaconline.org)^18^ during the 2013–2014 academic year. We examined respondents who self-identified as being in postgraduate years (PGYs) 1–3. The University of Michigan Institutional Review Board granted exemption to this study.

### Self-assessment questions: attitude, preparedness, and behavior

We used 18 predidactic survey questions to assess residents' attitudes, perceived preparedness, and self-reported clinical practice behaviors in delivering care to culturally diverse patients.^11,17^ Domains covered included clinic volumes of patients from racial/ethnic minority groups and low-socioeconomic status (SES), attitudes about practicing among diverse populations, consideration of culture when providing care, self-assessment of cultural sensitivity and unconscious racial bias, self-assessment of preparedness to take care of sociocultural diverse patients, and self-rated behaviors in delivering patient- and family-centered care (Supplementary Appendix 1).

### Pretest questions for knowledge assessment

Twelve pretest questions were developed by internal medicine faculty with expertise in cross-cultural care, health and healthcare disparities, curriculum development, and web-based learning. Clinical vignettes and questions were modeled on existing resources.^1,3,7,10,16,19–23^ The questions covered a range of topics in cross-cultural care, including caring for patients with language barriers, limited literacy, low-SES, and those from differing racial and ethnic minority groups. Questions were categorized as definition/recall, comprehension, and/or application (Supplementary Appendix 2). One question was not studied because of poor item discrimination.

### Study variables

Residency program characteristics included the program's classification as university based, community based, or military based. Programs regional location in the United States was grouped into one of four regions: Mid-West, West, South, or East (Supplementary Appendix 3).^24^ Programs were classified as being in either an urban (location in a city/town with a population of 50,000) or nonurban location (population less than 50,000).^25^ PGYs 1–3 level was used to categorize the residents' year of training. Resident knowledge of cross-cultural care that is focused on health and healthcare disparities was represented by the percentage of correct responses for both the overall pretest and the limited pretest composed of the seven questions that were focused on comprehension and application.

The set of questions related to attitude, preparedness, and behavior were assessed for correlation and reliability. Principal components analysis (PCA) was conducted on the five attitude, six preparedness, and five behavior items, respectively, to assess for correlation. Cronbach's alpha was used to measure the reliability of the items within each set of questions. After confirmation of correlation and reliability, three scales were created: attitude, preparedness, and behavior. The scales were created by summing up the corresponding question's response (each rated 0–100) within each set and then divided by 100. The scales were made to combine correlated information rather than having multiple variables measuring similar characteristics. For example, a high rating for the attitude scale represented an overall more positive attitude toward cross-cultural care.

### Main outcomes

The main outcome was the self-reported preparedness scale that measured the residents' perceived preparedness to care for patients from sociocultural diverse backgrounds.

### Secondary outcomes

A secondary outcome was knowledge of cross-cultural care with focus on health and healthcare disparities, as represented by the residents' percentage of correct responses for the overall pretest.

### Statistical analysis

Descriptive statistics were used to assess the percentage of residents' program classification, regional designation, location in an urban or nonurban setting, and the resident PGY level. Responses for all self-assessment questions were placed on a rating scale of 0 (low)–100 (high). Pretest responses were dichotomized to correct or incorrect and then tabulated for a composite score that was presented as the percentage of correct responses. A one-way ANOVA was used to compare pretest scores between the residents' three PGY levels. Linear regression analysis was performed to explore the association between preparedness with resident attitude, knowledge, clinical practice composition (of patients from racial and ethnic minority backgrounds and lower SES), PGY level, and residency program characteristics: program type, regional designation, and location in an urban or nonurban setting. A second linear regression model was used to explore associations between the residents' knowledge assessment score with their PGY level, attitude, and preparedness. Statistical analyses were performed using Stata/LP, version 13.^26^

## Results

### Characteristics of study population

Eighty-four out of 158 internal medicine residency programs (52.8%) participated in 29 states across the United States. Of the 1074 respondents who completed the module, 954 identified as being PGY-1, -2, and -3. The remaining 120 respondents were 10 PGY-4 and PGY-5 residents, 7 attending physicians, and 86 self-identified “other” respondents.

The majority of residents came from a university-based program, 619 (64.9%), followed by community-based program, 331 (34.7%), then military-based program, 4 (0.42%). The majority of residents trained in institutions that were located in an urban setting, 900 (94.3%) versus nonurban, 54 (5.7%). The Mid-West was the location for 288 (30.2%) residents, followed by 248 (26.0%) in the East, 236 (24.7%) in the South, and 182 (19.1%) in the West. There were 304 (31.9%) PGY-1 residents, 286 (30.0%) PGY-2 residents, and 364 (38.2%) PGY-3 residents (Table 1). The difference in overall scores on the pretest questions based on PGY was not statistically significant (PGY-1: 56% correct; PGY-2: 58% correct; PGY-3: 55% correct; *p*=0.08), and also did not differ on grouped questions requiring comprehension and application of knowledge (PGY-1: 51% correct; PGY-2: 55% correct; PGY-3: 51% correct; *p*=0.06).

**Table 1.** **Characteristics of Resident Participants**

Characteristic	*N* (%)
Residency program type	
Community	331 (34.7)
University	619 (64.9)
Military	4 (0.42)
Urban vs. nonurban location	
Nonurban	54 (5.7)
Urban	900 (94.3)
Region of United States	
East	248 (26.0)
Mid-West	288 (30.2)
South	236 (24.7)
West	182 (19.1)
Postgraduate level (PGY)	
PGY-1	304 (31.9)
PGY-2	286 (30.0)
PGY-3	364 (38.2)

### Correlation and reliability testing: attitude, preparedness, and behavior scales

The results from the PCA and Cronbach's alpha confirmed that the creation of the three scales (attitude, behavior, and preparedness) captured the intended information from the respective sets of questions. The PCA yielded one factor with eigenvalues >1.00 and all respective items were found to load on one component, which confirmed the correlation of the respective set of questions. The Cronbach's alpha for items related to attitude, behavior, and preparedness was 0.80, 0.86, and 0.90, respectively.

### Multiple linear regression outcomes

In the regression model, a residents' attitude toward cross-cultural care was positively associated with preparedness (beta coefficient [β]=0.57, 95% confidence interval [CI]: 0.49–0.64, *p*<0.001). Both residents who reported serving a large number of racial/ethnic minorities and low-SES patients were positively associated with preparedness (β=0.90, 95% CI: 0.56–1.24, *p*<0.001 and β=0.74, 95% CI: 0.37–1.10, *p*<0.001, respectively). The mean preparedness level was higher for residents who were at the PGY-2 and PGY-3 levels than those at the PGY-1 level (β=0.16, 95% CI: 0.02–0.31, *p*=0.03 and β=0.18, 95% CI: 0.05–0.32, *p*=0.01, respectively). Training at a university-based program was negatively associated with preparedness compared with training at a community-based program (β=−0.15, 95% CI: −0.28 to −0.02, *p*=0.03; Table 2).

**Table 2.** **Estimated Associations of Resident and Residency Program Characteristics with Self-Reported Preparedness (*N*=954)**

Characteristic	Beta-coefficient	95% CI	*p*
Attitude scale^a,b^	0.57	0.49 to 0.64	<0.001
Knowledge score (limited)^b,c^	−0.03	−0.11 to 0.06	0.57
Knowledge score (overall)^b,d^	−0.01	−0.08 to 0.05	0.73
Serves a large number of racial/ethnic minority patients^b^	0.90	0.56 to 1.24	<0.001
Serves a large number of patients with a low-socioeconomic status^b^	0.74	0.37 to 1.10	<0.001
Postgraduate level			
PGY-1	Reference		
PGY-2	0.16	0.02 to 0.31	0.03
PGY-3	0.18	0.05 to 0.32	0.01
Residency program type			
Community	Reference		
University	−0.15	−0.28 to −0.02	0.03
Military	0.73	−0.17 to 1.64	0.11
Region of United States			
East	Reference		
Mid-West	−0.05	−0.10 to 0.21	0.49
South	−0.06	−0.23 to 0.10	0.46
West	−0.17	−0.36 to 0.01	0.06
Urban vs. nonurban location			
Nonurban	Reference		
Urban	0.06	−0.19 to 0.31	0.63

Self-preparedness scale consisted of five validated questions that were grouped into a scale.

^a^ The attitude scale consisted of five validated questions that were grouped into a scale.

^b^ All continuous variables were rescaled by dividing by 100 for ease of interpretation purposes.

^c^ Knowledge score for the comprehension pretest questions only.

^d^ Knowledge score for overall pretest questions.

In the second linear model, behavior was negatively associated with knowledge (β=−0.40, 95% CI: −0.60 to −0.20, *p*<0.001). Among residents with average preparedness, there was a significant positive relationship between attitude and knowledge (β=0.51, 95% CI: 0.32–0.70, *p*<0.001). A significant interaction between the residents' attitude level and preparedness was observed. As the level of preparedness increased, the positive association between attitude and knowledge decreased (β=−0.15, 95% CI: −0.22 to −0.08, *p*<0.001; Table 3).

**Table 3.** **Estimated Association of Behaviors, Attitudes,^a^ and Self-Reported Preparedness^b^ with Knowledge of Disparities^c^ Among Internal Medicine Residents (*N*=954)**

Characteristic	Beta-coefficient	95% CI	*p*
Self-reported behavioral scale^d,e^	−0.40	−0.60 to −0.20	<0.001
Attitude scale^a,e^	0.51	0.32 to 0.70	<0.001
Self-reported preparedness scale^b,e^	0.05	−0.10 to 0.21	0.51
Modification effect of self-reported			
preparedness on attitude	−0.15	−0.22 to −0.08	<0.001
Postgraduate level			
PGY-1	Reference		
PGY-2	0.30	−0.03 to 0.63	0.08
PGY-3	0.03	−0.28 to 0.34	0.87

^a^ The attitude scale consisted of five validated questions that were grouped into a scale.

^b^ Self-preparedness scale consisted of six validated questions that were grouped into a scale.

^c^ Knowledge score for overall pretest questions.

^d^ The behavioral scale consisted of five validated questions that were grouped into a scale.

^e^ All of the above scales were rescaled by dividing by 100 for ease of interpretation purposes.

## Discussion

Among residents studied, exposure to a greater volume of racial/ethnic minority and low-SES patients was associated with a more positive attitude toward cross-cultural care and greater perceived preparedness to deliver care to these populations. A positive attitude toward cross-cultural care was also associated with better knowledge of related concepts. However, this knowledge did not improve during residency training. Also, preparedness to deliver cross-cultural care was found to be lower at university-based training programs than at community hospitals.

We also found an inverse relationship between clinical practice behaviors in cross-cultural care and knowledge. The relationship between attitude and knowledge was found to be negatively impacted by the effect of preparedness as well. The implications of these findings were unclear and could be better understood with further analysis on a more granular level.

Our findings that residents had poor knowledge of healthcare disparity-related concepts across PGY levels suggest that these concepts are not being taught during residency. Previous studies have shown that a number of providers continue to be unaware of the extent of healthcare disparities both nationally and in their own patient practices.^10,27^ Incorporating disparities education into cross-cultural curricula has the potential to better equip residents to deliver more equitable care to an increasingly racial and ethnic diverse patient population.^10,14^

In the United States, residents care for a disproportionate share of racial and ethnic minority and low-SES patients, commonly at university-based programs.^28^ Yet, residents' self-reported preparedness to care for vulnerable patients was found to be lower for those training at university-based programs than for those training at community-based programs in this study. It was unclear whether these findings were more influenced by resident-, patient-, community-, or institutional-level factors.

Residents who expressed less positive attitudes toward cross-cultural care and reported limited exposure to vulnerable patient populations felt less prepared to provide cross-cultural care. Residents are largely composed of young adults whose beliefs and moral values are well established. Cross-cultural care training may only have meaningful impact on a self-selected segment of residents who are open to developing their level of understanding and empathy for patients' social, economic, and cultural ecological context that influences their health.^10^ Curriculum need assessments may potentially aid in development of training that engages residents and shifts their attitudes toward a more patient-centered approach to delivering care. In addition, as healthcare moves toward value-based care, trainees may be more incentivize to engage in cross-cultural care training.^29^

The findings in our secondary analysis were unclear. We found that residents who highly rated their clinical practice behaviors in caring for racial and ethnic minority and low-SES patients had lower scores on knowledge. Furthermore, we saw that residents' preparedness had a negative impact on the relationship between their attitude and knowledge. Taken together, these findings could suggest that residents who perceive themselves as prepared to deliver culturally sensitive care may lack awareness of their own knowledge gaps, because even in the setting of positive attitudes, they do not demonstrate increased knowledge. Objective assessments of cultural competence and better education during residency training can begin to address knowledge and potential performance gaps.

Our study had several limitations that could potentially affect its generalizability. First, the 52.8% response rate of participating programs represents residents from a selected group of training programs and may not fully represent residents at nonparticipating internal medicine residency programs. Also, program directors commonly select modules from the PEAC library for their residents to complete; some programs make the completion of modules compulsory, whereas others do not. However, our sample of programs was geographically diverse and included university-based and community-based hospitals, suggesting generalizability of results. We also relied on self-reported data regarding preparedness and clinical practice behavior, which may not be reflective of the residents' actual delivery or quality of care provided.^30^ Our study strengths included the use of validated questions,^11,16^ the creation of three unique scales related to attitude, preparedness (including care of low-literate and low-SES patients), and behavior with domain content different from previously created cross-cultural scales,^31^ and the examination of the interaction of attitude and preparedness on knowledge.

## Conclusion

Our study suggests that more training is needed to increase internal medicine residents' knowledge of cross-cultural care as it relates to health and healthcare disparities. This study speaks of the opportunity for programs to enhance how residents are trained to care for an ever increasing diverse patient population. Also, more research should be done to examine residents' perceived preparedness to deliver cross-cultural care at university-based programs, especially because these sites give service to large volumes of vulnerable populations. Our findings underline a need to better understand the role of residents' attitudes on preparedness to provide care for sociocultural diverse patients. Furthermore, it will be informative to explore how residents' actual provision of care for diverse patients relates to their attitude and knowledge of health and healthcare disparities.

## Supplementary Material

Supplemental data

Supplemental data

Supplemental data

## Abbreviations Used

PCA: principal components analysis
PGY: postgraduate year
SES: socioeconomic status
