# Supplementary material for: Residents' Attitude, Knowledge, and Perceived Preparedness Toward Caring for Patients from Diverse Sociocultural Backgrounds
Source: Health Equity. 2017 Feb 1;1(1):43–9. doi: 10.1089/heq.2016.0010 (PMC5586003; doi:10.1089/heq.2016.0010)
Supplement: Supplementary file 1 [file Supp_Appendix1.pdf]

Supplementary Data

Supplementary Appendix 1. Self-Assessment Questions

Rate your level of agreement with the following questions from 0 (low/disagree strongly) to 100 (high/agree strongly)	0–100
Practice setting	
I deliver care to a large number of racial/ethnic minority patients in my practice	
I deliver care to a large number of patients with a low-socioeconomic status in my practice	
Attitude	
It is important to me that I practice in a setting that has a racially and ethnically diverse patient population.	
It is important to me that I practice in a setting that has a socioeconomically diverse patient population.	
How important do you feel it is for physicians to consider the patient's culture when providing care?	
Unconscious biases about patients based on their race may affect the way I make decisions about their care without my realizing it.	
"I am culturally sensitive with my patients"	
Rate your level of agreement with the following questions from 0 (low/disagree strongly) to 100 (high/agree strongly)	0–100
Preparedness	
How prepared do you feel to care for patients:	
From cultures different from your own.	
With a socioeconomic status different from your own.	
With health beliefs or practices at odds with Western medicine.	
With a distrust of the U.S. healthcare system.	
With limited literacy.	
Who are members of racial and ethnic minorities.	
Rate your level of frequency with the following questions from 0 (low/disagree strongly) to 100 (high/agree strongly)	0–100
Behavior	
How often do you do the following things when seeing your patients:	
I ask patients and families to tell me about their own explanations of health and illness.	
I ask patients and families to tell me about their expectations for care.	
I recognize potential barriers to service that might be encountered by different people.	
I act to remove obstacles (e.g., lack of insurance and need for interpreter) for patients of different cultures when I identify such obstacles.	
I find ways to adapt my services to patients and family cultural preferences.	
