# Supplementary material for: Residents' Attitude, Knowledge, and Perceived Preparedness Toward Caring for Patients from Diverse Sociocultural Backgrounds
Source: Health Equity. 2017 Feb 1;1(1):43–9. doi: 10.1089/heq.2016.0010 (PMC5586003; doi:10.1089/heq.2016.0010)
Supplement: Supplementary file 2 [file Supp_Appendix2.pdf]

Supplementary Appendix 2: Pretest Questions

Choose the correct answer: (correct answers are in bold).

A "health disparity" is defined as:

(a) Differences in health linked to biology and genetics
(b) Differences in health linked to social disadvantage
(c) Differences in health linked to behaviors
(d) Differences in health linked to childhood illness

A 42-year-old African American woman with history of hypertension and chronic kidney disease is in the hospital with a hypertensive emergency. She is frequently admitted for hypertensive emergency, often with chest pain and/or acute renal failure. In review of her social history, you note that she did not complete high school. She is unemployed and does not have health insurance. Which of the following are risk factors for health disparities?

(a) Multiple comorbidities
(b) Low-educational attainment
(c) Noncompliance
(d) Younger age

The scenario in which health disparities did not exist is known as:

(a) Health equity
(b) Health determination
(c) Health biology
(d) Health opportunity

Your next patient in clinic is a 23-year-old African American male who presents as a new patient referral after being treated in the emergency department (ED) for a sore throat. He went to the ED because he lacked a primary care provider, and has no insurance. He is homeless, with no income. He gets meals at the local shelter. The feature of this vignette that will be the greatest determinant of his health would be:

(a) Poor geography of opportunity
(b) Racial residential segregation
(c) Social determinants of health
(d) Poverty

An 18-year-old Hispanic man with poorly controlled asthma lives in a low-income neighborhood that has a high amount of air pollution because of a near-by bus terminal. He is nonadherent with all of his prescribed inhalers that he receives from a free health clinic in his area. He has a 9th grade education. Of the following features of this case, which is considered an upstream social determinant of his health?

(a) His nonadherence to medication
(b) Regular exposure to bus fumes
(c) His access to a free health clinic
(d) His level of education

A 20-year-old Native American/American Indian woman without significant past medical history lives in a low-income neighborhood. She has a 10th grade education. She works at a low-wage factory job that requires a lot of heavy lifting. She has begun having frequent episodes of low-back pain and has been receiving care at her neighborhood clinic. Of the following, which is considered a downstream social determinant of health?

(a) Her ethnicity
(b) Her education level
(c) Her job
(d) Her access to healthcare

Supplementary Appendix 2: Pretest Questions (continued)

It has been shown that African Americans and Latinos are prescribed fewer analgesics than Caucasians for pain management. This is an example of a:

(a) Healthcare disparity
(b) Cultural outcome
(c) Societal determinant of health
(d) Health inequity

A 72-year-old Pakistani man with limited English proficiency and a history of seizure disorder and bronchiectasis is admitted to the hospital with pneumonia. Today he is showing clinical improvement. He states that he was recently diagnosed with bronchiectasis and would like to know more about the disease. Which of the following is/are a risk factor(s) for limited health literacy?

(a) Older age 65
(b) Limited English proficiency
(c) Compromised health status
(d) All of the above

Your next patient is a 38-year-old Puerto Rican man with history of hypertension and tobacco use. He lives in a low-income neighborhood marked with blight and high crime. What term best explains the relationship of his residential location, access to socioeconomic resources, and its impact on his health?

(a) Limited residential opportunity
(b) Limited geography of opportunity
(c) Low-neighborhood cohesion
(d) Low-neighborhood organization

The Whitehall study of British civil servants was a landmark study done in the 1960s that:

(a) Demonstrated the impact on race/ethnicity on health outcomes
(b) Demonstrated the impact of the work environment on health outcomes
(c) Demonstrated the impact of socioeconomic status on health outcomes
(d) Demonstrated the impact of health literacy on health outcomes

The excess mortality in Harlem study showed that an African American male born in Harlem was less likely to reach the age of 65 than a male born in:

(a) Finland
(b) Lebanon
(c) China
(d) Bangladesh
