# Supplementary material for: Residents' Attitude, Knowledge, and Perceived Preparedness Toward Caring for Patients from Diverse Sociocultural Backgrounds
Source: Health Equity. 2017 Feb 1;1(1):43–9. doi: 10.1089/heq.2016.0010 (PMC5586003; doi:10.1089/heq.2016.0010)
Supplement: Supplementary file 3 [file Supp_Appendix3.pdf]

Supplementary Appendix 3. Residency Program Regions Across the United States

EAST (Mid-Atlantic/New England)

The Mid-Atlantic region: Delaware, Maryland, New Jersey, New York, Pennsylvania, Puerto Rico, and Washington, DC
New England: Connecticut, Maine, Massachusetts, New Hampshire, Rhode Island, and Vermont

MID-WEST (Mid-West)

Illinois, Indiana, Iowa, Kansas, Michigan, Minnesota, Missouri, Nebraska, North Dakota, Ohio, Oklahoma, South Dakota, and Wisconsin

SOUTH

Alabama, Arkansas, Florida, Georgia, Kentucky, Louisiana, Mississippi, North Carolina, South Carolina, Tennessee, Texas, Virginia, and West Virginia

WEST (California-Hawaii/Mountain West/Northwest)

California-Hawaii

Mountain West: Arizona, Colorado, Nevada, New Mexico, Utah, and Wyoming
Northwest: Alaska, Idaho, Montana, Oregon, and Washington
